# DNA alterations in ovarian adult granulosa cell tumours: A scoping review protocol

**DOI:** 10.1371/journal.pone.0303989

**Published:** 2024-06-14

**Authors:** Sven Karstensen, Karsten Kaiser, Caroline Moos, Tim Svenstrup Poulsen, Kirsten Jochumsen, Claus Høgdall, Finn Lauszus, Estrid Høgdall

**Affiliations:** 1 Department of Womens’s Health, University of Southern Denmark, Sygehus Sønderjylland, Aabenraa, Denmark; 2 Department of Clinical Research, University of Southern Denmark, Sygehus Sønderjylland, Aabenraa, Denmark; 3 Department of Pathology, Molecular Unit, University of Copenhagen, Herlev Hospital, Herlev, Denmark; 4 Department of Gynecology, University of Southern Denmark, Odense University Hospital, Odense, Denmark; 5 Department of Gynecology, University of Copenhagen, Rigshospitalet, Copenhagen, Denmark; Western Michigan University School of Medicine: Western Michigan University Homer Stryker MD School of Medicine, UNITED STATES

## Abstract

**Background:**

Identifying and describing molecular alterations in tumors has become common with the development of high-throughput sequencing. However, DNA sequencing in rare tumors, such as ovarian adult granulosa cell tumor (aGCT), often lacks statistical power due to the limited number of cases in each study. Questions regarding personalized treatment or prognostic biomarkers for recurrence or other malignancies therefore still need to be elucidated. This scoping review protocol aims to systematically map the current evidence and identify knowledge gaps regarding DNA alterations, actionable variations and prognostic biomarkers in aGCT.

**Methods:**

This scoping review will be conducted based on Arksey and O’Malley’s methodological framework and later modifications by JBI Evidence Synthesis. The protocol complies with Preferred Reporting Items for Systematic Reviews and Meta-Analyses extension for scoping reviews. All original publications describing molecular alterations of aGCT will be included. The search will be performed in May 2024 in the following databases: MEDLINE (Ovid), Embase (Ovid), Web of Science Core Collection and Google Scholar (100-top ranked).

**Discussion:**

This scoping review will identify knowledge and gaps in the current understanding of the molecular landscape of aGCT, clinical trials on actionable variations and priorities for future research. As aGCT are rare, a possible limitation will be the small sample sizes and heterogenic study settings.

**Scoping review registration:**

The review protocol is registered at Open Science Framework under https://doi.org/10.17605/OSF.IO/PX4MF.

## Background

Ovarian adult granulosa cell tumor (aGCT) is a rare tumor arising from the ovarian stroma and accounts for approximately 2–5% of ovarian malignancies [1, 2]. Most aGCTs are diagnosed at an early stage and treated curatively with surgical resection. Unfortunately, approximately 10–20% relapse with advanced tumor spread, sometimes many years after initial diagnosis [3, 4]. In addition, aGCT patients have a lifetime increased risk for other cancers, primarily estrogen-sensitive cancers (i.e. breast and endometrial cancer) [5, 6]. The molecular landscape of breast and endometrial cancers has been extensively researched, yet no common driver for mutations in breast, endometrial and aGCT has been identified [7]. To our knowledge, there is only one case report examining DNA variants found in women with aGCT and concurrent endometrial cancer [8].

Optimal management of aGCT presents significant challenges. Apart from the tumor stage, no prognostic biomarkers are used for prediction of potential recurrences [9, 10]. There is a lack of evidence-based treatment options for recurrent aGCT, except further surgery, and limited experience with targeted therapy [11–16]. Finally, there is sparse information published about determining a woman’s risk of other cancer diagnoses after an aGCT is identified.

Since the development of high throughput sequencing (Next Generation Sequencing, NGS), studies have described the mutational landscape of aGCT to determine actionable and prognostic variations [17–21]. A missense mutation in *FOXL2* (c.402C>G; p.C134W) was reported in ~95% of aGCT [22–24]. However, despite being of value in diagnosing aGCT correctly, a *FOXL2* mutation still has limited clinical relevance [25, 26]. Although trunctating *KMT2D* mutations, *TERT* promoter mutations and pathogenic *TP53* variants have been reported in aGCT-cohorts, a pattern that describes prognostic markers is yet to be identified [17–20, 27–29]. Interestingly, a recent study reported an increased expression of genes with hormone signalling functions in aGCT [30].

Due to the tumour’s rarity, molecular studies on aGCT are naturally limited to a small number of cases or extensive cross-sectional studies. These study designs cannot answer critical questions about the linkage between the genomic landscape and prognostic or actionable targets. To our knowledge, none of the existing molecular variants in aGCT are targetable for personalized treatment. Although several reviews on aGCT exist [31–38], none have systematically mapped the current knowledge of DNA variants in aGCT.

This scoping review aims to systematically describe the DNA variations in aGCT and reference these variants with well-established genetic variant databases. By referencing variations with genetic variant databases, we can report on the variant’s effect on disease development and the potential for targeted therapies.

## Methods and design

This review will be conducted in accordance with the methodology outlined by Arksey and O’Malley [39], and amendments proposed by the Joanna Briggs Institute (JBI) [40]. The review protocol is registered at Open Science Framework under this link: https://doi.org/10.17605/OSF.IO/PX4MF. The scoping review will be developed in five stages [39]:

- Stage 1: Defining the research question.
- Stage 2: Identification of relevant studies.
- Stage 3: Selection of the studies.
- Stage 4: Organisation and tabulation of the data.
- Stage 5: Summarisation, compilation and documentation of the results.

This protocol was developed in accordance with the guidelines from the PRISMA Extension for Scoping reviews (PRISMA-ScR) checklist [41] (S1 and S2 Checklists).

### Stage 1: Identification of the research question

Inspiration for this review was elicited from a recently published scoping review on the molecular alterations in peritoneal mesothelioma (PeM) [42]. This scoping review identified common mutations in rare cancers and used MyCancerGenome.org as the reference for actionable targets or clinical trials [43]. MyCancerGenome.org, OnkoKB, ClinVar, COSMIC, ClinicalTrial.gov are databases that store information about oncogenic mutations, targetable mutations and ongoing clinical trials regarding targeted therapy [43–46]. These databases will also be used as a reference for any molecular alterations identified in this scoping review. This scoping review aims:

- To explore the DNA alterations associated with aGCT and give an overview of potential treatment possibilities
- To investigate if reported DNA variations predict risk of recurrence, aggressive disease or a risk of developing other primary malignant tumors
- To reference molecular alterations found in aGCT with MyCancerGenome.org, OnkoKB, ClinVar, COSMIC, ClinicalTrial.gov (May 2024)
- To identify knowledge gaps between DNA alterations of aGCT and prognosis potential

### Stage 2: Identification of the relevant literature

The authors have developed a set of inclusion criteria based on the ‘Population–Concept–Context (PCC)’ framework proposed by JBI [47].

**Search strategy.** The search strategy will consist of two search terms (granulosa cell tumour AND molecular alterations). Each element will consist of database-specific subject headings (MeSH), and free text words with relevant truncation and proximity operators. The search terms were inspired by similar search strings published in reviews from the Cochrane Library [16, 48]. An example of the search strategy for Medline (OVID) is presented in Box 1.

**Box 1. Search syntax.**

**Table:** 

1	Granulosa Cell Tumor/ or ((granulosa adj3 (cancer* or carcino* or tumo* or neoplasm*)) or call exner bod* or (folliculoma adj3 ovar*) or (neoplastic adj3 granulosa)).mp. or Sex Cord-Gonadal Stromal Tumors/ or (((sex cord or sexcord) adj3 (cancer* or carcino* or tumo* or neoplasm*)) or gyandroblastoma*).mp.
2	Transcription, Genetic/ or Promoter Regions, Genetic/ or Mutation/ or Germ-Line Mutation/ or sequence analysis, dna/ or sequence analysis, rna/ or dna mutational analysis/ or multilocus sequence typing/ or whole genome sequencing/ or exome sequencing/ or Gene Expression/ or gene expression profiling/ or rna-seq/ or Single-Cell Gene Expression Analysis/ or polymorphism, genetic/ or polymorphism, single nucleotide/ or Comparative Genomic Hybridization/ or Chromosome Aberrations/ or Gene Rearrangement/ or Genetic Testing/ or Genetic Markers/ or Translocation, Genetic/ or ((promoter adj3 region*) or mutation* or mutant* or ((gene or genetic or genes) adj3 (alter* or rearrang* or re-arrang* or transcript*)) or mutagen* or deletion* or (copy adj3 number* adj3 variat*) or (compar* adj3 genom* adj3 hybrid*) or ((DNA or gene* or single-nucleotid*) adj3 polymorphism*) or (chromosom* adj3 (abberat* or instabil* or abnormal* or anomal* or error* or defect*)) or ((genetic or gene or genome* or sequenc*) adj3 analys*) or ((protein* or DNA or gene*) adj3 expression*) or ((gene or genetic) adj3 (marker* or transloc* or screening or testing)) or ((germ-line or germline or somatic) adj3 mutation) or ((DNA or RNA) adj3 sequenc*) or (tumor adj3 mutational adj3 burden) or (oncological adj3 parameters)).mp.
3	1 and 2

The search strategy will be translated for Embase (Ovid), Web of Science and Google Scholar (100-top ranked) and reviewed with an information specialist in health sciences.

### Stage 3: Study selection

A summary of the PCC and inclusion and exclusion criteria is shown in Box 2.

**Box 2. Summary of PCC.**

**Table:** 

	Inclusion	Exclusion
P-population	Women diagnosed with aGCT	Studies with aGCT-cell lines
C-concept	Studies will be considered if they investigate germline and somatic DNA alterations in aGCT.	Studies exclusively investigating the *FOXL2* mutation
C-context	Original research (observational, cross-sectional cohort studies, case reports, case series)	Grey literature
PCC: Population-Concept-Context, aGCT: adult granulosa cell tumor

Only peer-reviewed original research focusing aGCT and molecular alterations will be included. The aGCT diagnosis must have been defined and validated by pathologists prior to molecular analysis. Only articles describing high throughput sequencing techniques of somatic and germline DNA variations in women with aGCT will be considered. There will be no language or publication date restrictions, and all studies matching our criteria published up until the search date will be considered. Studies with cell lines and targeted DNA sequencing limited to only the *FOXL2* variant known to be present in ~95% of aGCT will be excluded. Any reviews of molecular profiles of aGCT and the prognostic values of this information will be used to identify additional primary studies by applying forward and backwards citation searching.

Results will be imported into covidence [49] for screening, and any duplicates will be removed. Two reviewers will screen titles and abstracts, applying inclusion and exclusion criteria (Box 1). Any full-text articles excluded after screening will include the reasoning behind exclusion. A PRISMA flowchart will summarise the search, screening and identification process for relevant studies.Any disagreements will be solved by discussion, or if necessary, an experienced third author will make the final decision.

Screening and study selection will be performed in March 2024.

### Stage 4: Charting the data

**Fig 1:**
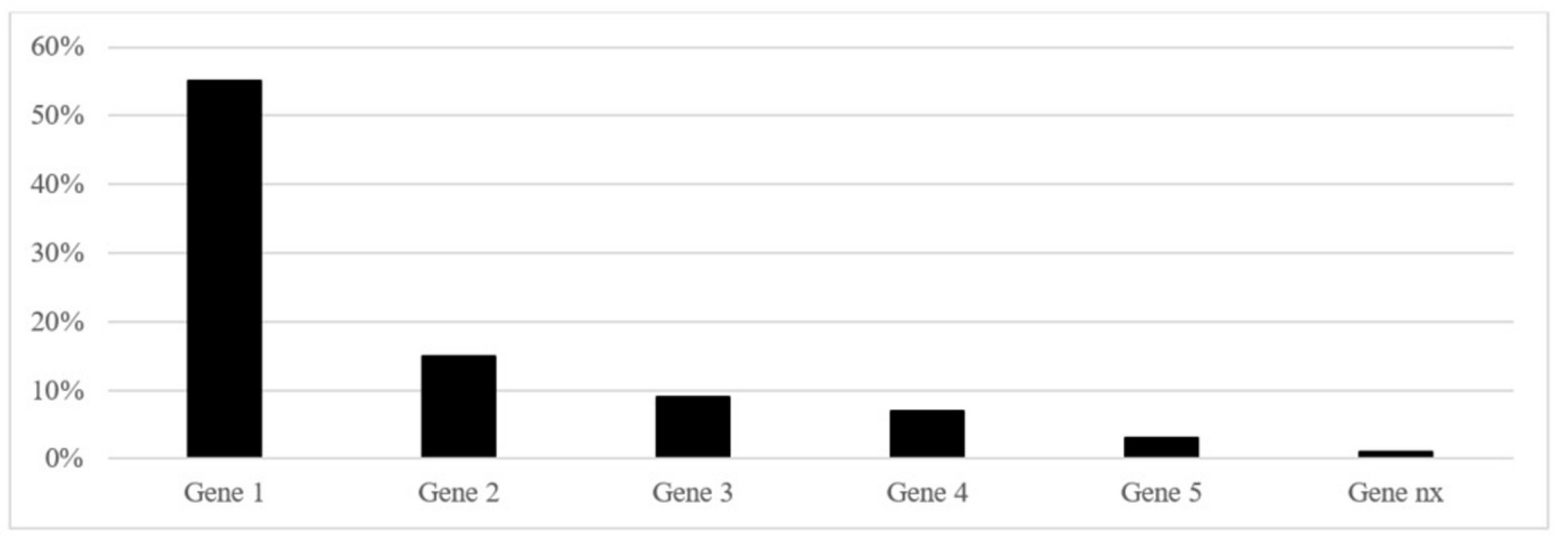
Suggested table to report gene alterations in >1% of the female adult granulosa cell tumor (aGCT) patients. N = XX.

The authors will use a chart or table (based on the JBI template source of evidence details, characteristics and results extraction instrument) to extract data blinded to each other [47]. Any somatic and germline DNA alterations, known as either pathogenic, likely pathogenic or of unknown significance according to OncoKB Cancer Gene List will be recorded [44]. Data to calculate frequencies will be plotted in STATA Release 17.0 (StataCorp, College Station, TX, USA). Relevant information will be presented in pre-planned tables (see Tables 1, 2 and 3 and Fig 1).

**Table 1:** Suggested charting form.

Reference	Year	Aim/purposes	Population and sample size	Sequencing technology	Extend of sequencing (WGS/WES/Panel)	Annotation software	Type of samples	Limitations/ advantages

WGS: Whole genome sequencing, WES: Whole exome sequencing

**Table 2:** Pathogenic variants in genes associated with an increased risk of other primary cancers.

Gene	Alteration	Variant classification (P, LP, VUS)	Frequency in aGCT	Associated risk of other primary cancer

aGCT, adult granulosa cell tumor; P, pathogenic; LP, likely pathogenic; VUS, variance of unknown significance.

**Table 3:** Genes with available targeted therapies.

Gene	Alteration	Variant classification (P, LP, VUS)	Frequency in aGCT	Targeted therapies type	Targeted drug

aGCT, adult granulosa cell tumor; P, pathogenic; LP, likely pathogenic; VUS, variance of unknown significance

Tables 2 and 3 will be generated by referencing any DNA pathogenic and likely pathogenic alterations with established databases (MyCancerGenome.org, OnkoKB, ClinVar, COSMIC, ClinicalTrial.gov) to identify the associated risks of other neoplasms and the possibility of targeted therapies. These proposed tables are preliminary and may be amended as the scoping review progresses.

### Stage 5: Collating, summarizing and reporting results

The study’s outcome will be published as a scoping review article containing texts, flow charts and tables. The flow chart will present the search strategy and study selection results. The identification and findings of the selected studies will be elaborated on in the article’s discussion section. Moreover, the research questions will be addressed based on these main findings. Finally, limitations, knowledge gaps and areas requiring further research will be highlighted.

### Patient and public involvement

No patient involvement.

## Discussion

The proposed scoping review article will systematically map the current knowledge about the DNA alterations in aGCT and the associated clinical impacts of these alterations. Therefore, the focus will be on pathogenic and likely pathogenic variations, as these may impact treatment options and disease development. Only one common missense mutation in *FOXL2* (c.402C>G; p.C134W) has been found in ~95% of aGCT [22–24]. Apart from this mutation, reported somatic variants are heterogenous, and little is known about the clinical impact of these variants in aGCT [18, 20]. Collating the results of studies will identify common and recurrent molecular DNA variants describing sub-groups of patients with different risk profiles. This review allows for the comparison of results across publications to identify new common variants. This comparison will assist in identifying knowledge gaps and priorities for clinical trials on actionable variations in aGCT. In addition, any DNA alterations eligible for personalized treatment, including immunotherapy, might be identified by referencing the alteration with well-established databases (MyCancerGenome.org, OnkoKB, ClinVar, COSMIC, ClinicalTrial.gov).

This scoping review methodology was previously used to map the molecular landscape in other rare tumors [42, 50, 51]. Limitations of this scoping-review approach include the broad scope and heterogeneity of studies reviewed. Studies may lack complete mutational data or clinical outcomes such as morbidity and mortality, hampering interpretation. Although NGS encompasses targeted gene-panel sequencing, whole exome and whole genome sequencing, each technique has strengths and limitations. Any conclusions from studies based on different molecular techniques should be cautious in interpretation.

## Supporting information

S1 Checklist PRISMA-P (Preferred Reporting Items for Systematic review and Meta-Analysis Protocols) 2015 checklist: Recommended items to address in a systematic review protocol. (DOC)

S2 Checklist Preferred Reporting Items for Systematic reviews and Meta-Analyses extension for Scoping Reviews (PRISMA-ScR) checklist. (DOCX)

## List of abbreviation

aGCT: adult granulosa cell tumor
NGS: next generation sequencing
PeM: Peritoneal Mesothelioma
JBI: Joanna Briggs Insitute
PRISMA: Preferred Reporting Items for Systematic Reviews and Meta-Analyses
PCC: Population-Concept-Context
