# Supplementary material for: DNA alterations in ovarian adult granulosa cell tumours: A scoping review protocol
Source: PLoS One. 2024 Jun 14;19(6):e0303989. doi: 10.1371/journal.pone.0303989 (PMC11178167; doi:10.1371/journal.pone.0303989)
Supplement: S1 Checklist — (DOC) [file pone.0303989.s001.doc]

**S1 Document. PRISMA-P (Preferred Reporting Items for Systematic review and Meta-Analysis Protocols) 2015 checklist: recommended items to address in a systematic review protocol***

| Section and topic | Item No | Checklist item |  |
| --- | --- | --- | --- |
| ADMINISTRATIVE INFORMATION | | |  |
| *Title:* |  |  |  |
| *Identification* | 1a | DNA alterations in ovarian adult granulosa cell tumours: a scoping review protocol | x |
| *Update* | 1b | No | x |
| *Registration* | 2 | Open Science Framework under this link: https://doi.org/10.17605/OSF.IO/PX4MF | x |
| *Authors:* |  |  |  |
| *Contact* | 3a | Sven Karstensen (corresponding author), Dept. of Womens’s Health, University Hospital of Souther Denmark, e-mail: [sven.hoedt.karstensen@rsyd.dk](mailto:sven.hoedt.karstensen@rsyd.dk), Kresten Phillipsensvej 15, 6200 Aabenraa, Denmark  Karsten Kaiser, Dept. of Womens’s Health, University Hospital of Souther Denmark, e-mail: [karsten.kaiser@rsyd.dk](mailto:karsten.kaiser@rsyd.dk)  Caroline Moos, Dept. of Clinical Research, University Hospital of Southern Denmark, e-mail: [caroline.margaret.moos@rsyd.dk](mailto:caroline.margaret.moos@rsyd.dk)  Tim Svenstrup Poulsen, Dept. of Pathology, Molecular Unit, Herlev Hospital, University of Copenhagen, e-mail: tim.svenstrup.poulsen@regionh.dk  Kirsten Jochumsen, Dept. of Gynecology, Odense University Hospital, e-mail: [kirsten.jochumsen@rsyd.dk](mailto:kirsten.jochumsen@rsyd.dk)  Claus Høgdall, Dept. of Gynecology, Rigshospitalet, University of Copenhagen, e-mail: [claus.hogdall@regionh.dk](mailto:claus.hogdall@regionh.dk)  Finn Lauszus, Dept. of Women’s Health, University Hospital of Southern Denmark, e-mail: finn.lauszus@rsyd.dk  Estrid Høgdall, Dept. of Pathology, Molecular Unit, Herlev Hospital, University of Copenhagen, e-mail: [estrid.hoegdall@regionh.dk](mailto:estrid.hoegdall@regionh.dk) | x |
| *Contributions* | 3b | SK, TSP and EH conceptualised the scoping review. SK drafted the protocol. CM reviewed the methodology and search syntax. KK, KJ, EH, CH and FL reviewed the manuscript for intellectual content. | x |
| *Amendments* | 4 | No ammendments | x |
| *Support:* |  |  |  |
| *Sources* | 5a | No funding | x |
| *Sponsor* | 5b | No sponsor | x |
| *Role of sponsor or funder* | 5c | N/A | x |
| *INTRODUCTION* | | |  |
| *Rationale* | 6 | Adult granulosa cell tumor is a rare tumor of the ovary. The tumor biology and microenvironment has been investigated more frequently the past years. However, cohorts lack power and reporting on clinical outcome is diverse or lacking in the published works. A scoping review approach is best suited to map the current information and to identify knowledge gaps. | x |
| *Objectives* | 7 | This scoping review aims to explore existing knowledge about the DNA alterations of ovarian aGCT. | x |
| *METHODS* | | |  |
| *Eligibility criteria* | 8 | Only peer-reviewed original research focusing on women diagnosed with aGCT and reporting DNA alterations of aGCT will be included. The aGCT diagnosis must have been defined and validated by pathologists prior to molecular analysis. All genetic and molecular testing of somatic and germline mutations in women with aGCT will be considered. There will be no language or publication date restrictions, and all studies matching our criteria published up until the search date will be considered. Studies with cell lines and targeted DNA sequencing limited to FOXL2 variants will be excluded. | x |
| *Information sources* | 9 | Embase, MEDLINE, Web of Science and Google Scholar (100-top ranked) searched November 1st 2023. |  |
| *Search strategy* | 10 | Search from MEDLINE (Ovid):  Granulosa Cell Tumor/ or ((granulosa adj3 (cancer* or carcino* or tumo* or neoplasm*)) or call exner bod* or (folliculoma adj3 ovar*) or (neoplastic adj3 granulosa)).mp. or Sex Cord-Gonadal Stromal Tumors/ or (((sex cord or sexcord) adj3 (cancer* or carcino* or tumo* or neoplasm*)) or gyandroblastoma*).mp. AND Transcription, Genetic/ or Promoter Regions, Genetic/ or Mutation/ or Germ-Line Mutation/ or sequence analysis, dna/ or sequence analysis, rna/ or dna mutational analysis/ or multilocus sequence typing/ or whole genome sequencing/ or exome sequencing/ or Gene Expression/ or gene expression profiling/ or rna-seq/ or Single-Cell Gene Expression Analysis/ or polymorphism, genetic/ or polymorphism, single nucleotide/ or Comparative Genomic Hybridization/ or Chromosome Aberrations/ or Gene Rearrangement/ or Genetic Testing/ or Genetic Markers/ or Translocation, Genetic/ or ((promoter adj3 region*) or mutation* or mutant* or ((gene or genetic or genes) adj3 (alter* or rearrang* or re-arrang* or transcript*)) or mutagen* or deletion* or (copy adj3 number* adj3 variat*) or (compar* adj3 genom* adj3 hybrid*) or ((DNA or gene* or single-nucleotid*) adj3 polymorphism*) or (chromosom* adj3 (abberat* or instabil* or abnormal* or anomal* or error* or defect*)) or ((genetic or gene or genome* or sequenc*) adj3 analys*) or ((protein* or DNA or gene*) adj3 expression*) or ((gene or genetic) adj3 (marker* or transloc* or screening or testing)) or ((germ-line or germline or somatic) adj3 mutation) or ((DNA or RNA) adj3 sequenc*) or (tumor adj3 mutational adj3 burden) or (oncological adj3 parameters)).mp.  Similar searches are executed on Embase, Web of Science and Google Scholar | x |
| *Study records:* |  |  |  |
| *Data management* | 11a | Covidence systematic review software (Veritas Health Innovation, Melbourne, Australia. Available at [www.covidence.org](http://www.covidence.org/).) will be used to screen and review literature. | x |
| *Selection process* | 11b | Two independent reviewers will screen the title and abstract. Disagreements will be resolved by discussion; if necessary, an experienced third reviewer will make the final decision. Full text review will be performed in the same manner. | x |
| *Data collection process* | 11c | Data extraction will be done by two independent reviewers. Initially a piloting form will be used (Tabel 1) to extract data and amendments will be taken if necessary. | x |
| *Data items* | 12 | Population: women with granulosa cell tumor, Concept: DNA variations (somatic or germline). Variables recorded: Reference, year of publication, aim/purpose, populations (age, tumor stage), sequencing methods, gene panel, software, type of sample | x |
| *Outcomes and prioritization* | 13 | This is a scoping review and no outcomes are necessary. | x |
| *Risk of bias in individual studies* | 14 | This is a scoping review and there will be no risk of bias assessment. | x |
| *Data synthesis* | 15a | DNA alterations will only be quantitatively assessed in proportions (frequency of alteration per case of adult granulosa cell tumor) | x |
|  | 15b | No statistical tests will be performed. | x |
|  | 15c | N/A | x |
|  | 15d | N/A | x |
| *Meta-bias(es)* | 16 | Differences in DNA sequencing will be appropriately addressed. |  |
| *Confidence in cumulative evidence* | 17 | This is a scoping review. Cumulative evidence will be reported in proportions and should only be used in reference to future research in this area. | x |

*** It is strongly recommended that this checklist be read in conjunction with the PRISMA-P Explanation and Elaboration (cite when available) for important clarification on the items. Amendments to a review protocol should be tracked and dated. The copyright for PRISMA-P (including checklist) is held by the PRISMA-P Group and is distributed under a Creative Commons Attribution Licence 4.0.**

*From: Shamseer L, Moher D, Clarke M, Ghersi D, Liberati A, Petticrew M, Shekelle P, Stewart L, PRISMA-P Group. Preferred reporting items for systematic review and meta-analysis protocols (PRISMA-P) 2015: elaboration and explanation. BMJ. 2015 Jan 2;349(jan02 1):g7647.*
