# Supplementary material for: DNA alterations in ovarian adult granulosa cell tumours: A scoping review protocol
Source: PLoS One. 2024 Jun 14;19(6):e0303989. doi: 10.1371/journal.pone.0303989 (PMC11178167; doi:10.1371/journal.pone.0303989)
Supplement: S2 Checklist — (DOCX) [file pone.0303989.s002.docx]

**S2 Document. Preferred Reporting Items for Systematic reviews and Meta-Analyses extension for Scoping Reviews (PRISMA-ScR) Checklist**

| **SECTION** | **ITEM** | **PRISMA-ScR CHECKLIST ITEM** | **REPORTED ON PAGE #** |
| --- | --- | --- | --- |
| **TITLE** | | | |
| Title | 1 | DNA alterations in ovarian adult granulosa cell tumours: a scoping review | 1 |
| **ABSTRACT** | | | |
| Structured summary | 2 | **Background**: With the development of high-throughput sequencing, determining the molecular alterations in tumors has become frequent. However, DNA sequencing in rare tumors, such as ovarian adult granulosa cell tumor (aGCT), often lacks power due to the limited number of cases in these studies. Therefore, questions about actionable targets for immunotherapy or prognostic biomarkers for women at high risk of recurrence or developing other malignancies remain unanswered. This scoping review protocol aims to systematically map the current evidence and identify knowledge gaps regarding DNA alterations, actionable variations and prognostic biomarkers in aGCT.  **Methods**: This scoping review will be conducted based on Arksey and O'Malley’s methodological framework, considering the later modifications made by JBI Evidence Synthesis. The protocol complies with Preferred Reporting Items for Systematic Reviews and Meta-Analyses extension for scoping reviews. All original publications regarding the molecular alterations of aGCT will be included. The intended search will will be performed in March 2024 in the following databases: MEDLINE (Ovid), Embase (Ovid), Web of Science Core Collection and Google Scholar (100-top ranked).  **Results**: Pending  **Conclusion**: Pending | 2 |
| **INTRODUCTION** | | | |
| Rationale | 3 | Adult granulosa cell tumor is a rare tumor of the ovary. The tumor biology and microenvironment has been investigated more frequently the past years. However, cohorts lack power and reporting on clinical outcome is diverse or lacking in the published works. A scoping review approach is best suited to map the current information and to identify knowledge gaps. | 3 |
| Objectives | 4 | This scoping review aims to explore existing knowledge about the DNA alterations of ovarian aGCT. | 3 |
| **METHODS** | | | |
| Protocol and registration | 5 | The review protocol is registered at Open Science Framework under this link: https://doi.org/10.17605/OSF.IO/PX4MF .  The protocol is also submitted for publication. | 4 |
| Eligibility criteria | 6 | Only peer-reviewed original research focusing on women diagnosed with aGCT and reporting molecular alterations of aGCT will be included. The aGCT diagnosis must have been defined and validated by pathologists prior to molecular analysis. All genetic and DNA sequencing of somatic and germline mutations in women with aGCT will be considered. There will be no language or publication date restrictions, and all studies matching our criteria published up until the search date will be considered. Studies with cell lines, and targeted DNA sequencing limited to FOXL2 variants will be excluded. | 6 |
| Information sources* | 7 | Embase, MEDLINE, Web of Science and Google Scholar (100-top ranked) searched November 1^st^ 2023. | 6 |
| Search | 8 | Search from MEDLINE (Ovid):  Granulosa Cell Tumor/ or ((granulosa adj3 (cancer* or carcino* or tumo* or neoplasm*)) or call exner bod* or (folliculoma adj3 ovar*) or (neoplastic adj3 granulosa)).mp. or Sex Cord-Gonadal Stromal Tumors/ or (((sex cord or sexcord) adj3 (cancer* or carcino* or tumo* or neoplasm*)) or gyandroblastoma*).mp. AND Transcription, Genetic/ or Promoter Regions, Genetic/ or Mutation/ or Germ-Line Mutation/ or sequence analysis, dna/ or sequence analysis, rna/ or dna mutational analysis/ or multilocus sequence typing/ or whole genome sequencing/ or exome sequencing/ or Gene Expression/ or gene expression profiling/ or rna-seq/ or Single-Cell Gene Expression Analysis/ or polymorphism, genetic/ or polymorphism, single nucleotide/ or Comparative Genomic Hybridization/ or Chromosome Aberrations/ or Gene Rearrangement/ or Genetic Testing/ or Genetic Markers/ or Translocation, Genetic/ or ((promoter adj3 region*) or mutation* or mutant* or ((gene or genetic or genes) adj3 (alter* or rearrang* or re-arrang* or transcript*)) or mutagen* or deletion* or (copy adj3 number* adj3 variat*) or (compar* adj3 genom* adj3 hybrid*) or ((DNA or gene* or single-nucleotid*) adj3 polymorphism*) or (chromosom* adj3 (abberat* or instabil* or abnormal* or anomal* or error* or defect*)) or ((genetic or gene or genome* or sequenc*) adj3 analys*) or ((protein* or DNA or gene*) adj3 expression*) or ((gene or genetic) adj3 (marker* or transloc* or screening or testing)) or ((germ-line or germline or somatic) adj3 mutation) or ((DNA or RNA) adj3 sequenc*) or (tumor adj3 mutational adj3 burden) or (oncological adj3 parameters)).mp.  Similar searches are executed on Embase, Web of Science and Google Scholar | 9 |
| Selection of sources of evidence† | 9 | Results will be imported to Covidence to screen relevant publications by two reviewers. Disagreements will be resolved by discussion; if necessary, an experienced third reviewer will make the final decision. | 7 |
| Data charting process‡ | 10 | The authors will use a charting form (based on the JBI template source of evidence details, characteristics and results extraction instrument) to extract data blinded to each other. Relevant information will be presented as suggested in Table 1, 2, 3 and Figure 1. | 7, 10 and appendix |
| Data items | 11 | Reference, year of publication, aim/purpose, populations (age, tumor stage), sequencing methods, gene panel, software, type of sample | 10 |
| Critical appraisal of individual sources of evidence§ | 12 | Not applicable, since we only include studies with well defined methods of DNA or RNA sequencing of aGCT. | N/A |
| Synthesis of results | 13 | Data will be plotted in STATA version 17 to handle and store information. Frequencies of alterations are calculated. | 7 |
| **RESULTS** | | | |
| Selection of sources of evidence | 14 | N/A.  This review is in progress. | Click here to enter text. |
| Characteristics of sources of evidence | 15 | N/A.  This review is in progress. | Click here to enter text. |
| Critical appraisal within sources of evidence | 16 | N/A.  This review is in progress. | Click here to enter text. |
| Results of individual sources of evidence | 17 | N/A.  This review is in progress. | Click here to enter text. |
| Synthesis of results | 18 | N/A.  This review is in progress. | Click here to enter text. |
| **DISCUSSION** | | | |
| Summary of evidence | 19 | N/A.  This review is in progress. | Click here to enter text. |
| Limitations | 20 | A possible limitation is the small sample size available as aGCT are rare. | 8 |
| Conclusions | 21 | N/A.  This review is in progress. | Click here to enter text. |
| **FUNDING** | | | |
| Funding | 22 | This research received no specific grant from any funding agency in the public, commercial or not-for-profit sectors. | 8 |

JBI = Joanna Briggs Institute; PRISMA-ScR = Preferred Reporting Items for Systematic reviews and Meta-Analyses extension for Scoping Reviews.

* Where *sources of evidence* (see second footnote) are compiled from, such as bibliographic databases, social media platforms, and Web sites.

† A more inclusive/heterogeneous term used to account for the different types of evidence or data sources (e.g., quantitative and/or qualitative research, expert opinion, and policy documents) that may be eligible in a scoping review as opposed to only studies. This is not to be confused with *information sources* (see first footnote).

‡ The frameworks by Arksey and O’Malley (6) and Levac and colleagues (7) and the JBI guidance (4, 5) refer to the process of data extraction in a scoping review as data charting*.*

§ The process of systematically examining research evidence to assess its validity, results, and relevance before using it to inform a decision. This term is used for items 12 and 19 instead of "risk of bias" (which is more applicable to systematic reviews of interventions) to include and acknowledge the various sources of evidence that may be used in a scoping review (e.g., quantitative and/or qualitative research, expert opinion, and policy document).

*From:* Tricco AC, Lillie E, Zarin W, O'Brien KK, Colquhoun H, Levac D, et al. PRISMA Extension for Scoping Reviews (PRISMAScR): Checklist and Explanation. Ann Intern Med. 2018;169:467–473. [doi: 10.7326/M18-0850](http://annals.org/aim/fullarticle/2700389/prisma-extension-scoping-reviews-prisma-scr-checklist-explanation).
