# Supplementary material for: DNA alterations in ovarian adult granulosa cell tumours: A scoping review protocol
Source: PLoS One. 2024 Jun 14;19(6):e0303989. doi: 10.1371/journal.pone.0303989 (PMC11178167; doi:10.1371/journal.pone.0303989)
Supplement: Attachment — Submitted filename: Response to reviewers.docx [file pone.0303989.s003.docx]

# To the editor

# Thank you for your reply and the constructive comments. We have dealt with question raised in the following response to the reviewers.

Even though we intended to explore the RNA variations, we have decided that presenting results on both DNA and RNA variations would be too broad and heterogeneous for one publication. Therefore, we have narrowed our scope to DNA variations.

**Reviewer #1: This scoping review protocol aims to systematically map the current evidence and identify knowledge gaps regarding molecular alterations, actionable variations and prognostic biomarkers in aGCT. This scoping review protocol is not an article paper, nor does it include specific experimental methods. In addition, there is a need to increase the accumulation of references in the introduction and discussion. The paper is inadequate and incomplete in its discussion of granulosa ovarian cancer.**

Answer: Thank you for reviewing our protocol manuscript. We agree that information about granulosa cell tumor was missing in the introduction. We have elaborated on the current information and the need for a scoping review. We did not aim at performing any experimental method. We aimed to define our scoping review method and publish our intended workflow to strengthen the validity of our results.

For the discussion section we have elaborated on the strength and limitations of a scoping review and to what a scoping review will add to the knowledge of DNA variations in granulosa cell tumors. We are not able to discuss specific results on DNA variants in granulosa cell tumors until we have finished the review.

**Reviewer #2: The manuscript presents a scoping review for a planned summary of previously published molecular sequencing data in ovarian granulosa cell tumors. Although this document is a detailed summary of a planned research methodology, there is no original research presented here.**

Answer: Thank you for reviewing our protocol manuscript. To our knowledge, there are no published papers on scoping review protocol regarding the molecular landscape of adult granulosa cell tumors. The existing reviews on aGCT are very informative. However, there is a lack of transparency in the methodology with missing descriptions on how the publications were gathered for the review. We expect a risk of missing relevant publications. We wish to provide transparency and reproducibility by publishing our review protocol.

**Reviewer #3: It is a very important area of study that the authors are conducting/reviewing, but I have some minor comments, which are described below.**

**1. Kindly state which reference manager you are using before importing eligible studies to Covidence for screening. you should also state the plan that you are using to remove duplicates.
2. Better the charting form include limitations of the included studies.
3. Stating "Animal studies " as an exclusion criterion is not necessary since the title of the objective is explaining that the review will be limited to human research specifically "women. "
4. Better to include "opinion papers and conferences" using "grey literature" as an umbrella since your review considers only published primary studies.**

Answer: Thank you for reviewing our manuscript. We have amended accordingly:

1: We have used EndNote as a reference manager. However, in the review process we have only used Covidence and Covidence has removed duplicates. This information is added.

2: In Table 1 we have added a column describing the strengths and limitation of the included studies.

3: Thank you for pointing out this fallacy. We have deleted this.

4: We agree and have changed this accordingly.
