# Supplementary material for: DNA alterations in ovarian adult granulosa cell tumours: A scoping review protocol
Source: PLoS One. 2024 Jun 14;19(6):e0303989. doi: 10.1371/journal.pone.0303989 (PMC11178167; doi:10.1371/journal.pone.0303989)
Supplement: Attachment — Submitted filename: Response to reviewers2.docx [file pone.0303989.s004.docx]

# To the editor

# Thank you for your reply.

**Reviewer #3: 1. The previous comments have been addressed but needs a few editorial and grammatical improvements like in Background "approx.": write it in full length" "Grey Litterature" in Box2. Avoid the second "t". Kindly check/correct throughout the document.**

Answer: Thank you for reviewing our protocol. We have revised the document carefully to correct language and grammatical errors.
